# Ambient air pollutants relate to hospital admissions for chronic obstructive pulmonary disease in Ganzhou, China

**DOI:** 10.11606/s1518-8787.2022056004324

**Published:** 2022-05-18

**Authors:** Xingye Zhou, Chenwei Li, Yanfang Gao, Chuanfei Zhou, Lei Huang, Xiaokang Zhang

**Affiliations:** I Gannan Medical University School of Public Health and Health Management Ganzhou China Gannan Medical University. School of Public Health and Health Management. Ganzhou, China

**Keywords:** Pulmonary Disease, Chronic Obstructive, epidemiology, Hospitalization, Air Pollution, adverse effects, Air Quality Standards, Models, Statistical

## Abstract

**OBJECTIVE:**

To evaluate the relationship between ambient air pollutants and chronic obstructive pulmonary disease in relatively low-polluted areas in China.

**METHODS:**

Atmospheric pollutants levels and meteorological data were obtained from January 2016 to December 2020. The medical database including daily hospital admissions for chronic obstructive pulmonary disease (ICD10: J44) was derived from the First Affiliated Hospital of Gannan Medical University. The generalized additive model was used to analyze the percentage change with 95% confidence interval in daily hospital admissions for chronic obstructive pulmonary disease associated with a 10 µg/m^3 ^increase in atmospheric pollutants levels.

**RESULTS:**

In total, occurred 4,980 chronic obstructive pulmonary disease hospital admissions (not including emergency department visits) during 2016–2020. The mean concentrations of daily PM_2.5_, PM_10_, SO_2_, NO_2_, O_3_, and CO were 37.5 μg/m^3^, 60.1 μg/m^3^, 18.7 μg/m^3^, 23.5 μg/m^3^, 70.0 μg/m^3^, and 1.2 mg/m^3^ in Ganzhou. Each 10 µg/m^3^ increment of PM_2.5_, PM_10_, NO_2_, and O_3_ were significantly associated with 2.8% (95%CI: 1.0–4.7), 1.3% (95%CI: 0.3–2.4), 2.8% (95%CI: 0.4–5.4), and 1.5% (95%CI: 0.2–2.7) elevation in daily chronic obstructive pulmonary disease hospital admissions. The estimates of delayed effects of PM_2.5_, PM_10_, NO_2_, and O_3_ were observed at lag6, lag6, lag8, lag1, respectively. The health effects of particulate pollutants (PM_2.5_ and PM_10_) may be independent of other pollutants. The adverse effects of air pollutants were more evident in the warm season (May–Oct) than in the cold season (Nov–Apr).

**CONCLUSION:**

Our study demonstrated that elevated concentrations of atmospheric pollutant (PM_2.5_, PM_10_, NO_2_, and O_3_), especially particulate pollutants, can be associated with increased daily count of hospital admissions for chronic obstructive pulmonary disease , which may promote further understanding of the potential hazards of relatively low levels of air pollution on chronic obstructive pulmonary disease and other respiratory disorders.

## INTRODUCTION

Chronic obstructive pulmonary disease (COPD), the third leading cause of morbidity and mortality in the world^1,2^, is characterized by a chronic inflammation of the lung that is not fully reversible and persistently limited airflow^3^. Because of the slow and partly irreversible progression of the disease, COPD is difficult to treat and can impose a significant financial burden on society^4^. Several articles indicated that ambient air pollutants exposure is a non-negligible risk factor for COPD. A study by Hwang et al.^5^ reported that particulate matter ≤ 2.5 µm in aerodynamic diameter (PM_2.5_) was positively associated with daily hospital admissions for acute exacerbation of COPD. Similarly, analyzing 73,076 hospital admission visits in Beijing, Gao et al.^6^ confirmed a 10 mg/m^3^ increase of PM_2.5_, particulate matter ≤ 10 µm in aerodynamic diameter(PM_10_), sulfur dioxide (SO_2_), nitrogen dioxide (NO_2_) and carbon monoxide (CO) concentration corresponded to a 0.8% ,0.9%, 2.1%, 3%, 6% increase in percent change in hospital admissions for COPD, respectively. In China, more attention was paid to air pollution prevention and control in the heavy pollution cities, such as Beijing, than in the low pollution cities, even though some articles indicated that the cities with less pollution showed greater risks of respiratory disorders^7,8^. Ganzhou is in the subtropical monsoon zone in the southern part of Jiangxi province. In 2020, the average daily concentration of PM_2.5_, PM_10_, SO_2_, NO_2_, ozone(O_3_), and CO in Ganzhou were 24 μg/m^3^, 41 μg/m^3^, 9 μg/m^3^, 17 μg/m^3^, 63 μg/m^3^, and 0.9 mg/m^3^, respectively, which are lower than the average concentration of 337 cities in China (33 μg/m^3^, 56 μg/m^3^, 10 μg/m^3^, 24 μg/m^3^, 138 μg/m^3^, and 1.3 mg/m^3^, respectively)^9^. Therefore, the cities with relatively low pollution levels, like Ganzhou, caught our attention.

To address the limitations of previous studies, we aimed to explore the association between ambient air pollutants and daily COPD hospital admissions in Ganzhou, China, from 2016 to 2020. Seasonal effects were also investigated.

## METHODS

### Data Collection

The data of COPD hospital admissions was obtained from First Affiliated Hospital of Gannan Medical University. Data elements of standardized electronic medical records include date of birth, date of hospital visit, gender, Chinese discharge diagnosis, 10th Revision (ICD-10) code, etc. Daily counts of hospital admissions for COPD (ICD-10 codes J44) were extracted from the medical database for the period of 2016–2020 (a total of 1,827 days). The health effects of exposure to atmospheric pollutants were evaluated by daily COPD hospital admissions.

The concentrations of air pollutants were measured by five fixed monitoring stations in Ganzhou, and the daily average concentrations at these monitoring stations were regarded as the daily level for each pollutant. In the study, we used daily means for PM_2.5_, PM_10_, SO_2_, NO_2_, and CO, and the daily maximum of 8-h means for O_3_. Furthermore, meteorological data, including relative humidity (%) and daily temperature (°C), were collected from Jiangxi Meteorological Bureau during the study period. No data for daily atmospheric pollutant concentration and meteorological characteristics are missing.

### Statistical Analysis

The database was constructed using Microsoft Excel, including daily mean concentrations of atmospheric pollutants, meteorological factors and daily hospital admissions for COPD. The SPSS software was used for statistical description and the ‘mgcv’ package in R was used for statistical analysis. Spearman’s correlation test was selected to explore the correlation between ambient air pollutants and meteorological factors.

Compared with the total urban population, daily COPD hospital admissions are a small probability event, which conforms to the Poisson distribution of the generalized additive model (GAM)^10,11^. Therefore, GAM was selected to assess the relationship between hospital admissions and atmospheric pollutants in this study. The model is as follows:


$$\log\left[E\left(Y_{t}\right)\right]=\alpha +\beta X_{t}+ns(\text{ time },df)+ns(\text{ Temp },df)+ns(RH,df)+DOW$$

E(Y_t_) indicates the expected daily count of hospital admissions on day t; α means a constant; β and X_t_ refer to the regression coefficient and the concentration of ambient air pollutant at day t, respectively; ns means natural spline function; df represent the degrees of freedom; DOW refers to the day of the week.

Several analyses were made to investigate the association between air pollution and hospital admissions for COPD. First, single-day lag models (lag0 to lag15) as well as cumulative lags models (lag01 to lag015) were selected to assess the effects of single atmospheric pollutant. Then, multi-pollutant models were used to check the effects of single air pollutant after adjustment by others. Finally, hospital admissions data were stratified by season to explore the relationship between atmospheric pollutants and COPD in different subgroups. Based on the local climate, November to April is considered the cold season and May to October the warm one.

In the study, the outcomes were described as the percentage change (PC) and 95% confidence interval (95%CI) of COPD hospital admissions with each 10 μg/m^3^ increase of atmospheric pollutant per day. Statistical significance was considered when p < 0.05.

## RESULTS

Table 1 shows the descriptive summary of daily COPD hospital admissions, air pollutants levels, and meteorological conditions. From January 1st, 2016 to December 31st, 2020, a total of 4,980 hospital admissions for COPD in Ganzhou, in which 81.6% were males and 70.4% were over 65 years old, formed the basis of our study. The annual number of the daily hospital admissions for COPD was 650 (13.1%), 648 (13.0%), 1,114 (22.4%), 1,502 (30.1%), and 1,066 (21.4%) for the five respective years (2016–2020). On average, occurred three hospital admissions per day due to COPD. During the study period, the daily mean concentrations (standard deviation) of PM_2.5_, PM_10_, SO_2_, NO_2_, O_3_ and CO were 37.5 (21.2) μg/m^3^, 60.1 (33.8) μg/m^3^, 18.7 (11.3) μg/m^3^, 23.5 (13.6) μg/m^3^, 70.0 (32.6) μg/m^3^, and 1.2 (0.3) mg/m^3^, respectively. The daily mean temperature was 19.7°C and relative humidity was 74.4%.

**Table 1:** Descriptive summary of daily COPD hospital admissions, atmospheric pollutants concentrations, and meteorological conditions.

	Mean ± SD	Minimum	P(_25_)	Median	P(_75_)	Maximum
COPD	2.7 ± 2.0	0	1	2	4	13
PM_2.5 _(μg/m^3^)	37.5 ± 21.2	6	23	33	47	197
PM_10 _(μg/m^3^)	60.1 ± 33.8	11	36	52	76	258
SO_2 _(μg/m^3^)	18.7 ± 11.3	2	11	16	23	73
NO_2 _(μg/m^3^)	23.5 ± 13.6	4	14	20	28	94
O_3 _(μg/m^3^)	70.0 ± 32.6	4	46	67	91	194
CO (mg/m^3^)	1.2 ± 0.3	0.6	1.0	1.2	1.4	2.9
Temperature (°C)	19.7 ± 8.1	0	13	21	27	32
Relative humidity (%)	74.4 ± 12.2	35	65	74	84	99

SD: standard deviation; P: percentile; COPD: chronic obstructive pulmonary disease; PM_2.5_: particulate matter ≤ 2.5 µm in aerodynamic diameter; PM_10_: particulate matter ≤ 10 µm in aerodynamic diameter; SO_2_: sulfur dioxide; NO_2_: nitrogen dioxide; O_3_: ozone; CO: carbon monoxide.

Table 2 shows the Spearman correlation between air pollutants and meteorological parameters. All the correlation coefficients between atmospheric pollutants and meteorological factors in this study were statistically significant. The levels of PM_2.5_, PM_10_, and SO_2_ were positively correlated with other air pollutant (correlation coefficient = 0.22–0.96), whereas O_3_ was negatively correlated with NO_2_, CO (correlation coefficient = -0.09 to -0.18). Temperature was negatively correlated with PM_2.5_, PM_10_, NO_2_, and CO (correlation coefficient = -0.13 to -0.39); however, it showed a significant positive correlation with SO_2_ and O_3_ (correlation coefficient = 0.07–0.37). Moreover, the relative humidity was negatively correlated with all the air pollutants (correlation coefficient = -0.08 to -0.65) except CO (correlation coefficient = 0.21).

**Table 2:** Spearman’s correlation between atmospheric pollutants and meteorological parameters.

	PM_2.5_	PM_10_	SO_2_	NO_2_	O_3_	CO	Temperature	Relative humidity
PM_2.5_	1							
PM_10_	0.96	1						
SO_2_	0.69	0.71	1					
NO_2_	0.62	0.66	0.51	1				
O_3_	0.26	0.33	0.22	-0.09	1			
CO	0.45	0.39	0.29	0.40	-0.18	1		
Temperature	-0.21	-0.13	0.07	-0.39	0.37	-0.32	1	
Relative humidity	-0.23	-0.36	-0.29	-0.08	-0.65	0.21	-0.32	1

PM_2.5_: particulate matter ≤ 2.5 µm in aerodynamic diameter; PM_10_: particulate matter ≤ 10 µm in aerodynamic diameter; SO_2_: sulfur dioxide; NO_2_: nitrogen dioxide; O_3_: ozone; CO: carbon monoxide.

Note: all correlation coefficients are statistically significant (p < 0.05).

**Figure 1:**
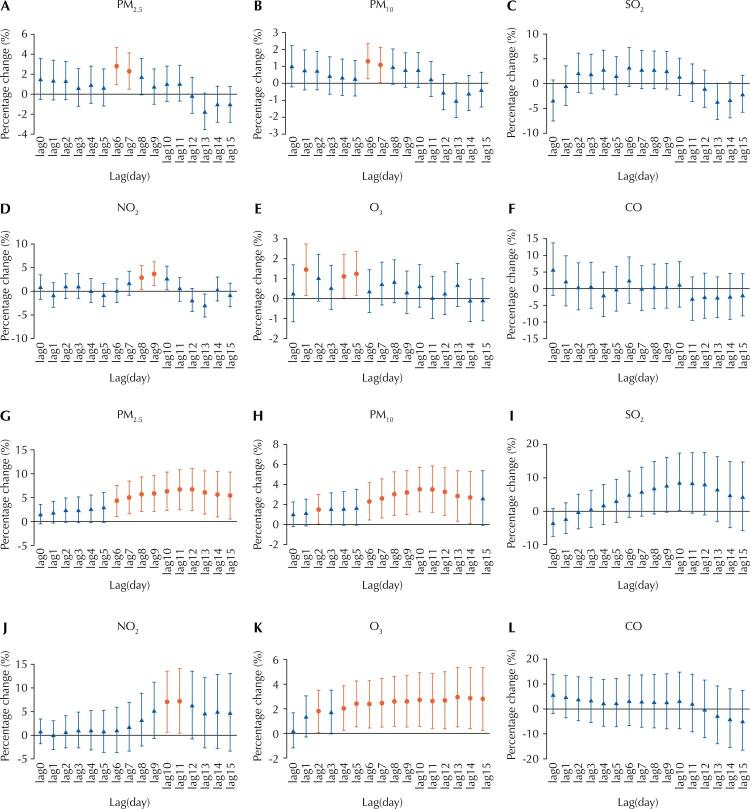
Percentage changes with 95%CI in hospital admissions for COPD with each 10 µg/m3 increase in atmospheric pollutants levels by single-day lag models (A ~ F) and cumulative lags models (G ~ L).

Figure 1 summarizes the analysis results in both single-day lag models (lag0 to lag15) and cumulative lags models (lag01 to lag015) for the percentage change in daily hospital admissions for COPD with per 10 μg/m^3^ increase of ambient air pollutants concentrations. The estimates using cumulative lags were higher than those using single-day lag. Overall, PM_2.5_, PM_10_, NO_2_, and O_3_ were positively related to COPD hospital admissions, while SO_2_ and CO showed no significant relationship in single-day lag models and cumulative lags models. In single-day lag model, the delayed effects of PM_2.5_ and PM_10_ were generally significant on lag6 and lag7, with the largest effect observed on lag6 (PM_2.5_: PC = 2.8%; 95%CI: 1–4.7; PM_10_: PC = 1.3%; 95%CI: 0.3–2.4). For NO_2_, the significant associations were observed on lag8 and lag9, and reached the maximum at lag9 (PC = 3.6%; 95%CI: 1.2–6.2). The single-day lag effects of O_3_ were significant on lag1, lag4, and lag5, with the largest effect observed on lag1 (PC = 1.5%; 95%CI = 0.2–2.7). In cumulative lags model, increases of 10 μg/m^3^ in the concentrations of PM_2.5_, PM_10_, NO_2_, and O_3_ were associated with increases of 6.6% (95%CI: 2.3–11.0), 3.5% (95%CI: 1.3–5.9), 7.1% (95%CI: 0.5–14.1), and 2.9% (95%CI: 0.6–5.4), respectively, in daily hospital admissions for COPD.

95%CI: 95% confidence interval; COPD: chronic obstructive pulmonary disease; PM_2.5_: particulate matter ≤ 2.5 µm in aerodynamic diameter; PM_10_: particulate matter ≤ 10 µm in aerodynamic diameter; SO_2_: sulfur dioxide; NO_2_: nitrogen dioxide; O_3_: ozone; CO: carbon monoxide.

Note: the lines in red color mean statistical significance.

The results of the multiple pollutant models are showed in Table 3. When adjusted by NO_2_ as well as O_3_, the association between particulate pollutants (PM_2.5_ and PM_10_) and COPD hospital admissions slightly changed and still showed statistical significance. After NO_2_ was controlled in the multi-pollutant model, the association between O_3_ and hospital admissions were relatively stable. Adjusted by O_3_, the association between NO_2_ and hospital admissions declined slightly, but still kept statistically significant. However, particulate pollutants weakened the effect of NO_2_ and O_3_, making them meaningless.

**Table 3:** Percent change (95%CI) in COPD hospital admissions in multi-pollutant models.

	Multi-pollutant models	Percent change	95%CI
PM_2.5_		6.6	(2.3–11.0)^a^
	+NO_2_	6.3	(1.5–11.6)^a^
	+O_3_	5.9	(0.9–11.1)^a^
PM_10_		3.5	(1.3–5.8)^a^
	+NO_2_	3.4	(0.8–6.4)^a^
	+O_3_	3.2	(0.6–5.5)^a^
NO_2_		7.1	(0.5–14.1)^a^
	+PM_2.5_	1.3	(-6.3–9.4)
	+PM_10_	1.6	(-7.4–11.3)
	+O_3_	6.8	(0.4–12.3)^a^
O_3_		2.9	(0.6–5.4)^a^
	+PM_2.5_	1.6	(-1.3–4.7)
	+PM_10_	2.0	(-1.0–5.2)
	+NO_2_	2.8	(0.5–5.4)^a^

95%CI: 95% confidence interval; COPD: chronic obstructive pulmonary disease.

^a^ p < 0.05

Note: the effects of multiday lag were used for daily COPD hospital admissions.

**Figure 2:**
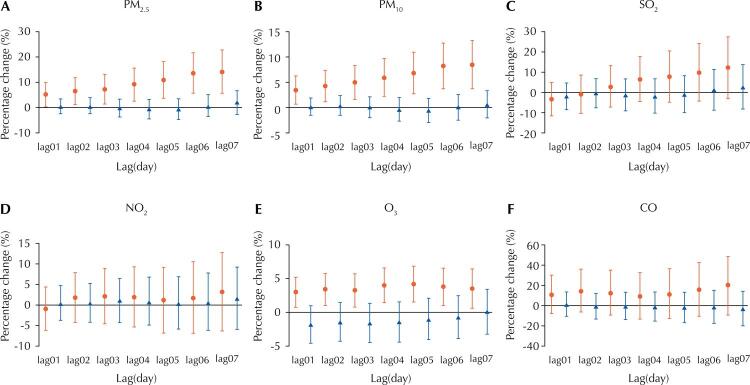
Percentage changes with 95%CIs in COPD hospital admissions with each 10 μg/m3 increase in PM2.5 (A), PM10 (B), SO2 (C), NO2 (D), O3 (E), and CO (F) concentrations, stratified by seasons.

As shown in Figure 2, PM_2.5_, PM_10_, and O_3_ were significantly associated with daily hospital admissions for COPD in the warm season (May–Oct). In contrast, the association between atmospheric pollutants and hospital admissions was not observed in the cold season (Nov–Apr). At lag07, each 10 μg/m^3^ increase in concentrations of PM_2.5_, PM_10_, and O_3_ corresponded to a 5.2% (95%CI: 0.5–10.0), 8.4% (95%CI: 3.8–13.3), and 3.5% (95%CI: 0.6–6.4) increase in daily COPD hospital admissions in the warm season, respectively.

95%CI: 95% confidence interval; COPD: chronic obstructive pulmonary disease; PM_2.5_: particulate matter ≤ 2.5 µm in aerodynamic diameter; PM_10_: particulate matter ≤ 10 µm in aerodynamic diameter; SO_2_: sulfur dioxide; NO_2_: nitrogen dioxide; O_3_: ozone; CO, carbon monoxide.

Note: November to April is considered the cold season (in blue color) and May to October is considered the warm season (in red color).

## DISCUSSION

This research is a quantitative assessment of the relationship between ambient air pollutants and the hospital admissions for COPD in Ganzhou in 2016–2020 using generalized additive models. In single-pollutant models, it was found that PM_2.5_, PM_10_, NO_2_, and O_3_ were positively correlated to COPD hospital admissions. Only PM_2.5_ and PM_10_ kept stable in multi-pollutant models. Additionally, the association between hospital admissions for COPD and ambient air pollutants were stronger in the warm season (May–Oct) than in the cold season (Nov–Apr).

Previous studies are consistent with our results, showing that atmospheric pollutants were linked with COPD. According to the study of Tian et al.^12^, for an interquartile range (IQR) increase in PM_2.5_, the daily hospital admissions for COPD increased by 6.0% (95%CI: 5.2–6.9) in the concurrent day. As found by Li et al.^13^ in their study on the effect of ambient fine particulate matter on hospitalizations in COPD: a 10 μg/m^3^ increase in PM_2.5_ was associated with a 3.1% (95%CI: 1.6–4.6) increase in COPD hospitalizations. Furthermore, Shin et al.^14^ studied data of Canada, and found that each positive association of COPD with PM_2.5_ increased the IQR in 3.4 μg/m^3^ (hazard ratio: 1.1). An ecological study conducted in Beijing, China, reported per 10 µg/m^3^ increments of the PM_10_ levels corresponded to 0.5% (95%CI: 0.3–0.7) increases in COPD hospital visits at lag0^6^. The lag pattern of PM_2.5_ and PM_10_ in our study is later than previous reports. This may indicate the visiting habit of patients may be limited by inadequate medical resources. Moreover, it might also be connected with environmental factors, including land-cover characteristics^15^.

Weichenthal et al.^16^ confirmed the relationship between the incidence of COPD and NO_2_. Another study covering the data from 218 Chinese cities also indicated that every 10 μg/m^3^ increase in NO_2_ was associated with a 1.7% (95%CI: 1.4–2.0) higher daily hospital admissions at the national-average level^17^. There already has toxicological study exploring the relationship between ozone and COPD. Wiegman et al.^18^ reported that ozone exposure in mice causes activation of oxidative pathways resulting in chronic bronchial inflammation and COPD. A systematic review by Gao et al.^19^ suggested that exposure to ozone was significantly positively associated with hospital admission for COPD. However, our results are inconsistent with several studies. For instance, a study conducted in Guangdong, China, found that concentrations of SO_2_ may be linked with the number of hospital admissions for COPD^20^. The research by Gao et al.^6^ showed that each 1 mg/m^3^ increase in CO was associated with a 5.0% (95%CI: 3.1–7.0) increase in COPD hospital admissions. The relationship between ambient air pollutants and hospital admissions for COPD from different regions may also vary. Such difference may be attributed to the distinct characteristics of pollutants, study areas and research populations. Besides, different cultural factors, demographic characteristics, and eating habits may all lead to different susceptibility of people in different regions.

Results of our research also indicated that the relationship between particulate pollutants (PM_2.5_ and PM_10_) and COPD hospital admissions kept robust in multi-pollutant models, which suggested the independent effects of particulate pollutants. Previous study has also provided similar results^21^. The effects of PM_2.5_ and PM_10_ were slightly reduced when NO_2_ and O_3_ were adjusted in the multi-pollutant model. A study by Liang et al.^22^ reported that the attenuation of particle effect can be interpreted by the collinearity between air pollutants. However, the collinearity between atmospheric pollutants makes it difficult to precisely assess the independent effects of single pollutant on inpatient visits of COPD. Therefore, the results of the combined effects of air pollutants should be interpreted with caution even when using multi-pollutant models^23^.

Our research showed that the estimated risk was higher in the warm season when compared with the cold one. The result is consistent with mostly relevant studies^6,12^. However, our findings conflicted with others indicating greater adverse effects in the cold season^5^. During the cold seasons, residents are more likely to close the window and stay indoors. While during the warm seasons, most people may increase the outdoor activity time^12^. Thus, more exposure to atmospheric pollutants and adverse effects in the warm seasons may be incurred. In the future, it is necessary to investigate the seasonal variation of particulate components in detail to better explore the estimates in different seasons.

This study has some strengths. First, our study was based on daily hospital admissions data, which can be more sensitive to reflect the association between COPD and ambient air pollutants. Second, this study adopted a model controlling a series of covariates to explore the relationship between COPD hospital admissions and atmospheric pollutants in a city with relatively low levels of air pollution. Nevertheless, several limitations should be considered. First, the design of our study is a descriptive study, which can provide hypotheses for other studies, but may be limited in causal inference. Second, we only used five fixed air monitoring stations as a proxy for personal exposure, so exposure measurement errors are inevitable^24^. Third, the data of hospital admissions were only collected from single city in China, so the generality of the results may be limited. Fourth, several influencing factors such as legal holidays and pandemic influenza are not included in the model, which may not fully reflect the relationship between air pollutants and COPD hospital admissions.

## CONCLUSIONS

In Ganzhou, China, there is a positive association between COPD and ambient air pollutants composed of PM_2.5_, PM_10_, NO_2_ and O_3_. The adverse effects of particulate pollutants (PM_2.5_ and PM_10_) were more stable. Additionally, atmospheric pollutants were more closely related to COPD in the warm season. We hope that our findings can help alert the government to pay more attention to the health effects of air pollutants in low-pollution areas.
